# Autonomy Support from Healthcare Professionals Improves Functioning in Early Psychosis Through Psychological Growth

**DOI:** 10.1093/schbul/sbaf249

**Published:** 2026-03-21

**Authors:** Helen Thai, Gillian A O’Driscoll, Richard Koestner, Emma Somer, Martin Lepage

**Affiliations:** Department of Psychology, McGill University, Montréal QC H3A 1G1, Canada; Douglas Research Centre, Montréal QC H4H 1R3, Canada; McLean Hospital, Harvard Medical School, Belmont, MA 02478, United States; Department of Psychology, McGill University, Montréal QC H3A 1G1, Canada; Department of Psychology, McGill University, Montréal QC H3A 1G1, Canada; Department of Psychology, McGill University, Montréal QC H3A 1G1, Canada; Department of Psychology, McGill University, Montréal QC H3A 1G1, Canada; Douglas Research Centre, Montréal QC H4H 1R3, Canada

**Keywords:** psychosis, schizophrenia, Self-Determination Theory, autonomy support, recovery

## Abstract

**Background:**

Early psychosis and schizophrenia-spectrum disorders are life-altering conditions, frequently associated with persistent impairments in functioning. Although evidence-based interventions exist, treatment outcomes remain variable. Self-Determination Theory (SDT) posits that autonomy-supportive environments facilitate positive outcomes; however, mediators of this association have been understudied in treatment settings of this clinical population.

**Study Design:**

Data were drawn from the Recovery After an Initial Schizophrenia Episode–Early Treatment Program study, a multisite, cluster-randomized controlled trial comparing NAVIGATE (*n* = 223; 78% male; *M*_age_ = 23.18), a coordinated specialty care intervention informed by SDT principles, to community care (CC; *n* = 180; 66% male; *M*_age_ = 23.08). Structural equation modeling was used to test whether perceived autonomy support from healthcare providers predicted overall functioning indirectly through recovery-oriented psychological growth. Models were evaluated separately by treatment condition and by study year, adjusting for baseline group differences.

**Study Results:**

Model fit indices indicated adequate fit (RMSEA≤.05, CFI > .95, SRMR<.08, TLI > .95). Among NAVIGATE participants, perceived autonomy support at 3 months predicted functioning at 12 months via recovery-oriented psychological growth processes at 6 months (ß = 1.41, SE = 0.73, *ab_ps_* = 0.063, 95%CI, 0.32-3.05); this indirect effect remained significant at Year 2. Significant full mediation effects were observed. No indirect effects were detected in the CC group.

**Conclusions:**

Treatment programs that emphasize autonomy-supportive principles, such as NAVIGATE, lead to improved functional outcomes by fostering recovery-oriented psychological growth. These findings highlight the relevance of SDT in clinical practice, providing a framework to optimize treatment outcomes by cultivating autonomy-supportive environments.

## Introduction

The first episode of psychosis (FEP) typically emerges in adolescence or young adulthood—critical periods that can profoundly alter life trajectory. Patients and families must navigate prognostic uncertainty, variable outcomes, and disruptions in psychosocial and occupational functioning.^1^ Although symptom severity^2–4^ and cognitive deficits^5^^,^^6^ predict prognosis, improvements in these domains do not consistently translate into better overall functioning, a key treatment goal in schizophrenia-spectrum disorders (SSDs).^7^ Identifying mediators may clarify *how* interventions are efficacious, particularly when effects on primary outcomes are indirect.

### Self-Determination Theory: Perceived Autonomy Support

Autonomy-supportive environments are increasingly recognized as central to promoting engagement and recovery in early psychosis.^8^ Within coordinated specialty care models like NAVIGATE, clinicians operationalize autonomy support through shared decision-making, motivational enhancement, and collaborative goal-setting. These practices are grounded in Self-Determination Theory (SDT), which posits that nurturing basic psychological needs (autonomy, competence, relatedness) foster internalization, sustained motivation,^9^^,^^10^ and well-being.^11^^,^^12^ In mental health contexts, SDT emphasizes the reciprocal relationship between individual agency and the treatment environment. Clinicians who provide rationale, validate lived experiences, and encourage reflection can enhance patients’ sense of volition and ownership over recovery, improving engagement and outcomes.^13–15^ Treatment settings thus present opportunities to apply autonomy-supportive principles.

### Autonomy Disruption in FEP and SSDs

The role of autonomy and autonomy support in psychosis is critically important yet often overlooked. While SSDs presents with significant heterogeneity, disruptions in autonomy are consistently recognized as central features.^10^ Such disruptions may manifest as difficulties in coherently integrating experiences, leading to diminished self-agency and impaired volitional control. For example, individuals with persecutory delusions may avoid social interactions or refuse assistance even when needed. These autonomy disruptions are shaped not only by symptoms, but also by systemic experiences (eg, involuntary hospitalization, forced medication, stigmatizing practices) that undermine patients’ capacity to seek help or make decisions.^16–18^ Traditional hierarchical treatment approaches that define success as compliance with externally prescribed interventions can further erode agency. Moreover, negative symptoms and cognitive deficits can make even basic tasks feel overwhelming and effortful, perpetuating helplessness, hopelessness, and disengagement from care. These processes highlight the need for treatment environments that deliberately restore autonomy.

### Recovery-Oriented Psychological Growth

Conceptualizations of recovery in serious mental illness have evolved from a focus on symptom remission to a broader emphasis on psychological growth, empowerment, and meaning.^19–21^ Recovery is a personal, nonlinear process characterized by renewed agency, hope, and self-efficacy—outcomes paralleling motivational mechanisms described in SDT. Autonomy-supportive healthcare environments facilitate this process by promoting internalization of values and self-determined goal pursuit.^9^^,^^10^ The Mental Health Recovery Measure (MHRM),^22^ a validated self-report instrument developed by service users, captures these processes through domains such as self-redefinition, empowerment, and goal striving. Prior work from the Recovery After an Initial Schizophrenia Episode Early Treatment Program (RAISE-ETP) demonstrated that autonomy support from clinicians is positively associated with psychological well-being and personal recovery across treatment phases.^23^ These findings suggest that psychological growth may represent a key pathway through which autonomy-supportive interventions enhance functional outcomes.

### The Present Study

Although SDT has demonstrated broad relevance to mental health treatment,^24–26^ applications in psychosis remain sparse^27^ and few studies have examined underlying mechanisms of change.^27^ The NAVIGATE program within RAISE-ETP^28^^,^^29^ provides a unique opportunity to evaluate these processes in a large multisite trial. NAVIGATE integrates shared decision-making, tailored psychotherapy, and coordinated pharmacologic and psychosocial supports, explicitly embedding SDT-consistent principles of autonomy support. Prior findings revealed that NAVIGATE participants reported greater perceived autonomy support and better functioning than community care (CC).^30^^,^^31^ Yet, the mechanisms underlying these improvements remain unexplored. Leveraging RAISE-ETP data, this study examined whether patients’ perceived autonomy support from providers predicted overall functioning via recovery-oriented psychological growth across the 2-year treatment period. We hypothesized that recovery-oriented psychological growth at 6 months would mediate the relation between autonomy support post-treatment initiation (3 months) and overall functioning at 12 months among NAVIGATE participants, and that this indirect effect would extend to Year 2. No mediation effects were anticipated for CC.

## Methods

### Participants and Study Design

Participant data were derived from the National Institute of Mental Health’s (NIMH) RAISE-ETP study, a longitudinal, multisite, cluster-randomized clinical trial. RAISE-ETP compared a comprehensive intervention (NAVIGATE) to standard CC. The trial enrolled 404 participants aged 15-40 years, with treatment centers randomly allocated to administer either the NAVIGATE program (*n* = 17 centers) or CC (*n* = 17 centers). Of these, 223 participants were assigned to the NAVIGATE program and 181 to the CC program. Participants were followed for study data collection over a 2-year period, with interventions provided for up to 2 years. Participants were eligible if they had a DSM-IV diagnosis of schizophrenia, schizoaffective disorder, schizophreniform disorder, brief psychotic disorder, or psychotic disorder not otherwise specified.^32^ All were required to have experienced just a single episode of psychosis and received 6 months or less of lifetime antipsychotic medication treatment. Eligibility was contingent upon the ability to undergo research assessments in English. Individuals with a diagnosis of affective psychosis, substance-induced psychotic disorder, or other serious medical conditions were excluded. All participants provided written informed consent, and those under 18 years of age provided assent alongside parental or guardian consent. Given that site-based personnel were not blinded to treatment allocation, the assessment strategy used both site-based and centralized evaluations to mitigate bias. Site-based assessments were conducted by trained research assistants, while centralized assessments were performed by evaluators with clinical experience and who were blinded to the treatment assignment and study design. Participating sites comprised a mix of community mental health programs and academic-affiliated clinics across the United States. Site procedures aligned with local clinical workflows, with research assessments standardized across sites as previously described. Enrollment occurred between July 2010 and July 2012, with the last participant completing 2 years of treatment in July 2014. Study assessments were paused during any periods of hospitalization or incarceration of participants but were resumed following their release or discharge. Detailed description of the recruitment procedures, clinical site selection, randomization process, treatment components, and study outcomes are reported elsewhere.^28^

### Interventions

NAVIGATE is a coordinated specialty care model that includes individualized medication management, family education, resilience-focused individual therapy, and supported employment and education services (intervention manuals can be found at https://www.navigateconsultants.org/manuals.html). In contrast to traditional symptom-focused approaches, NAVIGATE incorporates key principles of SDT, which posits that well-being and sustained motivation are fostered when individuals experience autonomy, competence, and relatedness.^12^ Core competencies for NAVIGATE team members were explicitly aligned with these needs: *Shared decision-making* supported autonomy by encouraging patients to participate as active partners in their treatment, rather than passive recipients of clinical directives; *Strengths and resiliency* promoted competence and relatedness by helping individuals identify meaningful goals, build self-acceptance, and develop fulfilling relationships; *Motivational enhancement* supported autonomous decision-making, particularly for individuals ambivalent about change, eliciting personally endorsed reasons for treatment and reinforcing sense of volition; *Psychoeducation* and *cognitive-behavioral interventions* further strengthened patients’ competence by scaffolding insight and skill acquisition in real world contexts; and *Collaboration with natural supports* (ie, individuals in regular contact with the patient outside of treatment) helped reinforce relatedness and continuity of care beyond the clinical setting. Treatment typically involved intensive interaction, often weekly, with team members for the first 6-12 months. This was followed by less frequent engagement, such as monthly meetings, for the next 12-18 months. NAVIGATE teams consisted of licensed prescribers and master’s- or doctoral-level clinicians. Prior to the study’s initiation, NAVIGATE personnel underwent specific training, and throughout the study, sites received ongoing consultation and fidelity monitoring to ensure the program’s integrity and model adherence. The control condition, Standard CC, involved treatment-as-usual at participating clinics and did not include SDT-informed training, supervision, or fidelity protocols beyond those necessary to support study follow-up.

### Measures

The analyses reported in this study focus on a subset of measures from the RAISE-ETP study. Thus, only these measures will be described in full. Baseline sociodemographic variables were self-reported and included age, sex, race, marital status, education, and living situation (Table 1).

**Table 1:** Participant Descriptive Statistics

	**NAVIGATE** **(*n* = 223)**		**Standard CC** **(*n* = 181)**	
	***N***	**%**	***N***	**%**
**Demographics**				
Sex				
Male	173	78	120	66
Female	50	22	61	34
Age (*M* ± SD)	23.18	5.21	23.08	4.90
Race				
White	138	62	80	44
Black	63	28	89	49
Other	22	10	12	7
Hispanic ethnicity	55	25	18	10
Marital status				
Currently married	14	6	10	6
Widowed/divorced/separated	14	6	8	4
Never married	195	87	163	90
Living situation				
Independent	40	18	32	18
Supported or structured	7	3	7	4
With family	158	71	129	71
Homeless or shelter	18	8	13	7
Highest level of education				
Some college or higher	71	32	54	30
Completed high school	75	34	58	32
Some high school	67	30	58	32
Some or completed grade school	9	4	11	6
Current student	35	16	47	26
Currently employed	28	13	30	17
**Clinical**				
DSM-IV Diagnosis				
Schizophrenia	113	51	101	56
Schizoaffective	43	19	38	21
Schizophreniform	43	19	24	13
Brief psychotic disorder	1	1	1	1
Psychotic disorder NOS	23	10	17	9
Prescribed one or more antipsychotic	182	82	155	86
Prior hospitalization(s)	169	76	147	81
	***M***	**SD**	***M***	**SD**
Duration of untreated psychosis (weeks)	178.91	248.73	211.43	277.49
Total PANSS score	78.32	14.95	74.54	14.87
**Overall functioning** (QLS total)				
Baseline	50.89	18.44	54.77	18.99
12 months	64.20	22.20	60.10	20.70
24 months	68.41	25.20	63.50	20.50
**Perceived autonomy support** (HCCQ)				
Baseline	5.59	1.08	5.48	1.37
3 months	5.86	1.11	5.42	1.36
12 months	5.90	1.13	5.49	1.25
**Recovery-oriented psychological growth** (MHRM)				
Baseline	73.70	17.90	72.80	19.40
6 months	76.50	18.10	74.60	18.10
18 months	77.90	17.90	74.90	18.10

Perceived Autonomy Support from healthcare professionals was assessed using the Health Care Climate Questionnaire (HCCQ).^33^^,^^34^ This questionnaire consists of 6 items, each rated on a 7-point Likert scale (1 = Strongly Disagree, 7 = Strongly Agree), to evaluate participants’ perceptions of autonomy support from their treatment team. Examples of items include “I feel that my clinicians have given me choices and options” and “My clinicians convey confidence in my ability to make changes.” Scores were averaged to derive a mean autonomy support score, with higher scores reflecting greater perceived autonomy support. Measurements were taken every 3 months, with the 3-month and 12-month scores used as predictors in the main analyses. Cronbach’s alphas for these measures were 0.90, 0.92, and 0.93 at baseline, 3 months, and 12 months, respectively.

Recovery-Oriented Psychological Growth was assessed using the MHRM,^22^ a validated self-report tool uniquely developed by service users, making it one of the few measures shaped directly by the experiences of those it intends to assess. This measure was designed to capture psychological processes that are of particular importance to service users themselves, reflecting the broader definition of recovery, beyond symptom management. Consequently, we chose to refer to the MHRM as “Recovery-oriented psychological growth,” recognizing that definitions of recovery are diverse and continually evolving. The MHRM includes items that tap into constructs such as overcoming stuckness (eg, “Even though there are hard days, things are improving for me”), discovering new potentials (eg, “I am making progress towards my goals”), self-empowerment (eg, “I have control over my mental health problems”), self-redefinition (eg, “Even though I may still have problems, I value myself as a person of worth”), and advocacy/commitment (eg, “I cope effectively with stigma associated with having a mental health problem”). The RAISE-ETP study used 15 of the full 30-item scale, rated on a 7-point Likert scale (The RAISE-ETP protocol administered a 15-item subset of the 30-item MHRM to reduce assessment burden while retaining coverage of core recovery processes (overcoming stuckness, new potentials, self-empowerment, self-redefinition, advocacy/commitment). Internal consistency in our sample was high at each timepoint (α = 0.92-0.93), supporting reliability of the administered subset.). Total scores could range from 15 to 105, with higher scores indicating better recovery-oriented psychological growth. Measurements were taken every 3 months, with scores from the 6- and 18-month assessments used as mediators in the main analyses. Cronbach’s alphas were 0.92, 0.93, and 0.93 for baseline, 6 months, and 18 months, respectively.

### Overall Functioning

Overall functioning was measured by the Quality of Life Scale (QLS),^35^ a semi-structured interview comprising 21 items rated on a 6-point Likert scale by centralized raters blinded to the study conditions. It assesses social interactions, instrumental role functioning, and engagement in regular activities. The total score, used as the main outcome in the principal RAISE-ETP study and this analysis, could range from 0 to 126, with higher scores indicating better functioning. Assessments were conducted biannually; the 12- and 24-month scores were used as treatment outcomes. Cronbach’s alphas were 0.86, 0.88, and 0.90 for baseline, 12 months, and 24 months, respectively.

Covariates included in the analysis were baseline measurements of the mediator (MHRM) and outcome (QLS) in our models^36^ and variables that showed significant baseline differences between the NAVIGATE and CC groups.^29^ Specifically, NAVIGATE participants had more males, higher PANSS total scores, and fewer individuals attending school compared to those in the CC group. These variables—biological sex (male = 0, female = 1), student status (yes = 1, no = 0), and PANSS total scores—were used to control for potential confounding effects in the treatment outcome analysis.

### Statistical Analyses

Descriptive statistics were used to characterize the sample at baseline. Pearson's correlations were conducted to examine relationships between key variables of interest. The main analyses focused on mechanisms of change through Structural Equation Modeling (SEM) mediation analyses. As reported in a previous study,^30^ results from the 3-level conditional linear growth model revealed that the NAVIGATE program was successful in increasing participants’ perceived autonomy support from healthcare workers over time, whereas no significant changes were detected for standard CC. Thus, separate group mediation models were conducted to test the direct and indirect effects of perceived autonomy support post-treatment initiation (*X*; measured by HCCQ at 3 and 12 months) on functional outcomes (*Y*; measured by QLS at 12 and 24 months), mediated by recovery-oriented psychological growth (*M*; assessed with MHRM at 6 and 18 months). Moreover, prior work revealed condition-specific trajectories in perceived autonomy support, suggesting non-equivalence of paths across groups.^37^^,^^38^ Although a single moderated-mediation model is more parsimonious, it assumes sufficient homogeneity of structural paths to be modeled jointly.^39^^,^^40^ Moreover, modeling each group separately permits examination of group differences without increasing the number of estimated parameters, a critical consideration when sample size is small relative to model complexity.^41^ Timepoint selection for mediation intended to reflect theoretical sequencing (ie, autonomy support early in each treatment year ➔ subsequent recovery-oriented psychological growth ➔ later functioning) and the availability/spacing of assessments. The models were tested separately in Year 1 and Year 2 using the relevant variables in each year. Although models were estimated separately by condition, we nonetheless included baseline group differences as covariates to address residual confounding and enhance comparability with prior RAISE-ETP analyses.

Model fit was assessed using standard indices (RMSEA, CFI, SRMR, TLI). Missing data were handled using Full Information Maximum Likelihood estimation. Direct effects were considered significant at *P* < .05, and percentile bootstrap confidence limits (1000 resamples) were used to determine the significance of indirect effects (95% CI excluding zero).^42^ Partially standardized mediated effect (*ab_ps_*) was employed to evaluate the effect size of the indirect effects, as simulation studies have observed that this measure exhibits lower bias and greater stability compared to the proportion and ratio mediated.^43^ This effect size measure (There are not yet guidelines for small, medium, and large partially standardized indirect effects, however the indirect effect (*ab_ps_*) are generally unbiased in single and multiple mediator models and have a clear interpretation.^43^) is interpreted as the expected change in standard deviations in *Y* (QLS) via the mediator (MHRM), for a 1-unit increase in *X* (HCCQ). Based on the main findings, post-hoc analyses using cross-lagged panel mediation models were conducted to establish temporal and causal inferences. However, these models demonstrated poor fit, likely due to measurement noninvariance, complexity from adding temporal dimensions that led to overfitting or underfitting, and/or an inadequate sample size to detect effects.^44^ Consequently, these findings are not included in the report. All analyses were conducted in R Studio version 2024.09.1 + 394 using the semTools and lavaan packages.^45^^,^^46^

### Data Transparency

This study involved a secondary analysis of publicly available data from RAISE-ETP, accessed through the NIMH Data Archive. A related article^30^ by other authors previously explored the role of autonomy support in treatment outcomes; however, the current study addresses distinct research questions that have not been examined in prior or forthcoming publications, to the best of our knowledge. Preliminary findings were presented in poster format at the 2025 Schizophrenia International Research Society conference in Chicago, Illinois, but this manuscript represents the first full-length publication of the study. All analyses adhered to the standards of the Journal Article Reporting Standards,^47^ including complete reporting of the sample size, all data exclusions (if any), manipulations, and measures. The dataset is de-identified and requires formal registration with the NIMH Data Archive; thus, raw data cannot be shared publicly due to ethical and privacy-related restrictions. This secondary analysis was not pre-registered and was exploratory with a priori theoretical expectations derived from SDT. The original trial was registered on ClinicalTrials.gov (NCT01321177).

## Results

### Descriptive Statistics

The study sample included 404 participants (NAVIGATE *n* = 223; CC *n* = 181), ages 15-40, predominantly young adults in the early course of illness with a mean age of 23. NAVIGATE participants had more males (NAVIGATE *n* = 173; CC *n* = 120) higher PANSS total scores (NAVIGATE *M* = 78.31, *SD* = 14.95; CC *M* = 74.54, *SD* = 14.87), and fewer individuals attending school (NAVIGATE *n* = 35; CC *n* = 47) compared to those in the CC group. Table 1 illustrates baseline descriptive statistics for each treatment group in detail. Table 2 contains correlations between major variables of interest across time for all participants. Overall, associations between autonomy support and recovery-oriented psychological growth were robust across time periods, with 26 out of 30 correlations positively significant (*P* < .05).

**Table 2:** Correlations Between Major Study Variables

	*M* (SD)	1	2	3	4	5	6	7	8	9	10	11	12	13	14	15	16	17
1. B Autonomy Support (AS)	5.55 (1.21)	–																
2. M3 AS	5.66 (1.24)	0.38^***^	–															
3. M6 AS	5.69 (1.21)	0.41^***^	0.63^***^	–														
4. M12 AS	5.66 (1.18)	0.33^***^	0.45^***^	0.54^***^	–													
5. M18 AS	5.68 (1.21)	0.26^***^	0.35^***^	0.43^***^	0.62^***^	–												
6. M24 AS	5.82 (1.21)	0.28^***^	0.32^***^	0.38^***^	0.57^***^	0.60^***^	–											
7. B Psychological Growth (PG)	73.26 (18.53)	0.37^***^	0.24^***^	0.28^***^	0.17^**^	0.12^*^	0.18^***^	–										
8. M3 PG	73.58 (18.49)	0.13^*^	0.33^***^	0.25^***^	0.18^**^	0.16^**^	0.18^**^	0.57^***^	–									
9. M6 PG	76 (18.26)	0.19^***^	0.30^***^	0.42^***^	0.18^**^	0.16^**^	0.21^***^	0.60^***^	0.68^***^	–								
10. M12 PG	77.44 (17.81)	0.20^**^	0.22^***^	0.28^***^	0.32^***^	0.21^***^	0.20^***^	0.54^***^	0.64^***^	0.70^***^	–							
11. M18 PG	78.07 (17.79)	0.13	0.20^***^	0.26^***^	0.34^***^	0.35^***^	0.35^***^	0.40^***^	0.45^***^	0.52^***^	0.54^***^	–						
12. M24 PG	79.72 (12.57)	0.23^***^	0.15	0.25^**^	0.24^***^	0.27^***^	0.42^***^	0.45^***^	0.49^***^	0.51^***^	0.50^***^	0.55^***^	–					
13. B Functioning	52.55 (18.84)	0.16^**^	0.11^*^	0.13^*^	0.06	0.03	0.00	0.20^***^	0.14^**^	0.17^**^	0.19^**^	0.13^*^	0.15^*^	–				
14. M6 Functioning	61.77 (21.46)	0.08	0.12	0.18^**^	0.08	0.10	0.08	0.13^*^	0.18^***^	0.27^***^	0.18^**^	0.12	0.21^***^	0.39^***^	–			
15. M12 Functioning	62.16 (21.08)	0.07	0.04	0.10	0.17^**^	0.07	0.09	0.10^*^	0.10	0.17^**^	0.27^***^	0.22^***^	0.21^**^	0.34^***^	0.41^***^	–		
16. M18 Functioning	63.43 (23.02)	−0.05	0.03	0.06	0.11	0.13^*^	0.12^*^	0.12^*^	0.11	0.20^**^	0.21^***^	0.42^***^	0.27^***^	0.44^***^	0.37^***^	0.49^***^	–	
17. M24 Functioning	65.12 (23.18)	0.08	0.16^**^	0.19^**^	0.15^*^	0.12	0.23^*^	0.09	0.14^*^	0.21^***^	0.16^**^	0.23^***^	0.40^***^	0.33^***^	0.38^***^	0.33^***^	0.48^***^	–

B = Baseline; M3, M6, M12, M18, M24 = Month 3, 6, 12, 18, and 24, respectively. Abbreviations: AS = Autonomy support; PG = [Recovery-oriented] psychological growth. ^*^P < .05, ^**^P < .01, ^***^P < .001.

### Main Analyses

Aligned with SDT and building on published RAISE ETP findings, we hypothesized that recovery-oriented psychological growth at 6 months would mediate the relationship between autonomy support post-treatment initiation (3 months) and overall functioning at 12 months exclusively for participants in the NAVIGATE group. We anticipated no mediation effects for the standard CC group. Similarly, for the NAVIGATE group, we expected that recovery-oriented psychological growth at 18 months would mediate the relationship between autonomy support at 12 months and overall functioning at 24 months, with no effects anticipated for the standard CC group. To account for baseline group differences, sex, student status, and PANSS symptom severity were included as covariates. Pre-treatment measurements of recovery-oriented psychological growth (mediator) and functioning (outcome) were included as covariates to control for potential confounding effects.^36^

SEM mediation assumptions were met,^41^ including no violations of multicollinearity, linearity, and homoscedasticity, and the use of bootstrapping allowed flexibility regarding the distribution of endogenous variables. Model fit indices indicated adequate fit: RMSEA≤.05, CFI > .95, SRMR<.08 TLI > .95.

**Figure 1:**
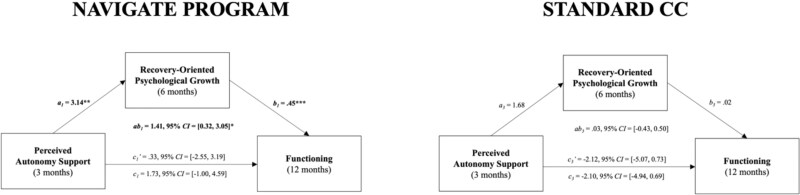
NAVIGATE Program vs. Standard CC Mediation Models in Year One. Mediation models representing the effect of perceived autonomy support on functioning via recovery-oriented psychological growth. *a* and *b* represent the 2 paths that make up the indirect path (*ab*), *c* represents the total effect of perceived autonomy support, and *c*’ represents the direct effect of perceived autonomy support controlling for recovery-oriented psychological growth. ^*^P < .05, ^**^P < .01, ^***^P < .001.

**Figure 2:**
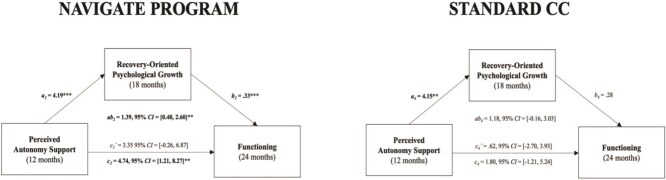
NAVIGATE Program vs. Standard CC Mediation Models in Year 2. Mediation models representing the effect of perceived autonomy support on functioning via recovery-oriented psychological growth. *a* and *b* represent the 2 paths that make up the indirect path (*ab*), *c* represents the total effect of perceived autonomy support, and *c*’ represents the direct effect of perceived autonomy support controlling for recovery-oriented psychological growth. ^*^P < .05, ^**^P < .01, ^***^P < .001.

NAVIGATE Mediation Models. For the NAVIGATE group, mediation analysis revealed that perceived autonomy support after treatment initiation was a significant predictor of recovery-oriented psychological growth at 6 months (*a*_1_), ß = 3.14 (SE = 1.11, *P* = .005, 95% CI, 1.12-5.42). Subsequently, recovery-oriented psychological growth at 6 months was a significant predictor of overall functioning at 12 months (*b*_1_), ß = .45 (SE = 0.14, *P* = .001, 95% CI, 0.18-0.75). Next, we examined total, indirect, and direct effects. The total effect (*c*_1_) of autonomy support at 3 months on functioning at 12 months was not significant, ß = 1.73 (SE = 1.43, *P* = .22, 95% CI, −1.00 to 4.59). The indirect effect (*ab_1_*) of autonomy support at 3 months on functioning at 12 months through recovery-oriented psychological growth at 6 months was statistically significant, ß = 0.1.41 (SE = 0.73, *ab_ps_* = .063, 95% CI, 0.32-3.05), while the direct effect (*c_1_’*) was not, ß = 0.33 (*SE* = 1.45, *P* = .82, 95% CI, −2.55 to 3.19), suggesting full mediation. These effects persisted into the second year of treatment: Autonomy support at 12 months was a significant predictor of recovery-oriented psychological growth processes at 18 months (*a*_2_), ß = 4.19 (SE = 1.14, *P* < .001, 95% CI, 2.12-6.47). Recovery-oriented psychological growth at 18 months, in turn, significantly predicted overall functioning at 24 months (*b*_2_), ß = 0.33 (SE = 0.09, *P* < .001, 95% CI, 0.14-0.52). The total effect (*c*_2_) of autonomy support at 12 months on functioning at 24 months was significant, ß = 4.74 (SE = 1.78, *P* = .008, 95% CI, 1.21-8.27). The indirect effect (*ab_2_*) of autonomy support at 12 months on functioning at 24 months through recovery-oriented psychological growth at 18 months was statistically significant, ß = 1.39 (SE = 0.55, *ab_ps_* = .055, 95% CI, 0.48-2.60). The direct effect (*c_2_’*) was not significant, ß = 3.35 (SE = 1.75, *P* = .055, 95% CI, −0.26 to 6.87), indicating full mediation. Together, results from the mediation models for NAVIGATE support our mediation hypotheses (Figures 1 and 2).

Standard CC Mediation Models. For the standard CC group, mediation analysis revealed that perceived autonomy support after treatment initiation did not predict recovery-oriented psychological growth processes at 6 months, (*a*_3_), ß = 1.68 (SE = 1.20, *P* = .16, 95% CI, −0.68 to 4.09), nor did recovery-oriented psychological growth at 6 months predict functioning at 12 months (*b*_3_), ß = .02 (SE = 0.11, *P* = .88, 95% CI, −0.22 to 0.21). No significant total (*c*_3_), ß = −2.10 (*SE* = 1.43, *P* = .14, 95% CI, −4.94 to 0.69), indirect (*ab_3_*), ß = 0.03 (SE = 0.22, *P* = .91, 95% CI, −0.43 to 0.50), and direct effects (*c_3_’*), ß = −2.12 (*SE* = 1.44, *P* = .14, 95% CI, −5.07 to 0.73), were detected for Year 1. In Year 2, perceived autonomy support at 12 months was a significant predictor of recovery-oriented psychological growth processes at 18 months (*a*_4_), ß = 4.15 (SE = 1.97, *P* = .035, 95% CI, 0.56-8.38) and recovery-oriented psychological growth at 18 months marginally predicted functioning at 24 months (*b*_4_), ß = 0.28 (*SE* = 0.16, *P* = .073, 95% CI, −0.00 to 0.61). The indirect effect (*ab_4_*) of autonomy support at 12 months on functioning at 24 months through recovery-oriented psychological growth at 18 months was not statistically significant, ß = 1.18 (SE = 0.80, *P* = .14, 95% CI, −0.16 to 3.03). Similarly, no significant total (*c*_4_), ß = 1.80 (SE = 1.64, *P* = .27, 95% CI, −1.21 to 5.24), or direct effects (*c_4_’*), ß = 0.62 (SE = 1.72, *P* = .72, 95% CI, −2.70 to 3.93), were detected. Thus, these null mediation results supported our hypotheses (Figures 1 and 2).

## Discussion

Our study leveraged the RAISE-ETP dataset^28^^,^^29^ to investigate the impact of perceived autonomy support from providers on functional improvements in early psychosis and SSDs, with recovery-oriented psychological growth serving as a mediator. Prior research in SDT applied to clinical settings,^10^^,^^48–52^ has reported that autonomy-supportive environments not only enhance perceived autonomy support but also predict improved treatment outcomes. We extend early psychosis literature by specifying *how* autonomy-supportive care relates to functional recovery, that is, via patient-reported recovery-oriented psychological change. Our findings suggest that structured embedding of autonomy support, as in NAVIGATE, may strengthen psychological growth mechanisms relative to CC, where such principles may be inconsistently applied.

NAVIGATE is one of few interventions that has effectively targeted and enhanced patients’ perceived autonomy support from providers undergoing FEP treatment.^30^ The link between autonomy support and positive treatment outcomes has been corroborated across studies on depression, eating disorders, and substance use.^13^^,^^50^^,^^51^ Situating our analysis within this broader literature, we highlight the transdiagnostic importance of autonomy support as a therapeutic process. Our study makes a novel contribution by examining *how* autonomy support may enhance treatment outcomes, revealing that recovery-oriented psychological growth plays a significant mediating role.

The mediation models conducted separately for each intervention year revealed nuanced patterns: In Year 1, the effect of autonomy support on functioning was fully mediated by recovery-oriented psychological growth, with no significant direct or total effect. In Year 2, there were both significant indirect and total effects, though the direct effect remained non-significant. These findings suggest that early autonomy support exerts cumulative impact, consistent with SDT and recovery-oriented care.

### Recovery-Oriented Psychological Growth as a Mediator

Recovery-oriented psychological growth, quantified with the MHRM, functions as a crucial mediator in the relationship between perceived autonomy support and overall functioning. Prior RAISE ETP work^30^ revealed higher perceived autonomy support was positively related to better functioning at baseline, 6, 12, and 18 months for NAVIGATE and at later timepoints for CC. Moreover, the strongest effects were detected toward the beginning of treatment for NAVIGATE and end of treatment for CC. Despite positive associations between autonomy support and treatment outcomes, our study revealed that the mediating effect of recovery-oriented psychological growth was prominently observed in NAVIGATE yet absent in standard CC. This divergence highlights how NAVIGATE’s structured SDT-informed practices (eg, collaborative goal-setting, motivational enhancement, strengths-based planning) activate psychological growth processes early in treatment, whereas the absence of such scaffolding in CC may limit or delay these effects.

### Autonomy Support in Treatment Settings

The stronger mediation effects observed in NAVIGATE suggests how autonomy support interacts with structured program elements. While CC providers may exhibit autonomy-supportive behaviors on an individual basis, NAVIGATE systematically embeds these principles across treatment modules and maintains fidelity through supervision and training. This design ensures consistency and reinforces autonomy support as a central therapeutic process rather than a clinician-dependent variable. In CC, the positive association between autonomy support and functional outcomes emerged later,^8^ perhaps reflecting cumulative patient-provider contact, whereby continued engagement in treatment allows patients to gradually perceive increased autonomy support that scaffolds psychological growth. This contrasts with NAVIGATE, where specific treatment elements (eg, shared decision making, self-determination though goal-setting, tailoring interventions to individual need) are designed to enhance autonomy support more immediately and independently of treatment duration.

We propose 2 reasons why early provision of autonomy support is critical. First, it helps retain patients in treatment from the outset, as evidenced by prior research revealing that participants in NAVIGATE were less likely to drop out compared to those in CC.^53^ Additionally, pattern-specific treatment effects^53^ between NAVIGATE and CC indicate that early dropouts in NAVIGATE might occur because these individuals benefitted significantly during the initial, more intense phases of treatment and chose to discontinue as their needs decreased. In contrast, dropouts in CC could stem from receiving insufficient treatment. Second, early provision of autonomy support is consistent with the phased reduction of therapeutic contact in NAVIGATE, which realistically mirrors the constraints and operational practices of public healthcare systems. Such practical considerations promote a design that focuses on early autonomy-support to optimize engage and efficiency—helping patients internalize self-regulatory skills that sustain recovery beyond active treatment. The absence of a direct effect and presence of an indirect effect suggest that the benefits of autonomy support operate through patients’ internal processes of psychological growth, which in turn contribute to improved functioning over time. In Year 2, persistence of significant indirect effects alongside total effects points to the continued importance of psychological growth that is scaffolded on autonomy-supportive engagement.

### Clinical Applications

Clinically, these findings highlight the importance of cultivating autonomy-supportive environments, both relationally and programmatically, within early psychosis services. Such services can foster autonomy support not only through clinician behaviors, but also through program-level structures that embed autonomy-supportive principles across care components. Programs like NAVIGATE exemplify this by integrating shared decision-making, family psychoeducation, and supported employment and education services within an autonomy-supportive framework. At both the systemic and individual levels, engaging patients in collaborative treatment planning, eliciting and validating personal values to enhance intrinsic motivation, and reinforcing competence through recognition of mastery-based progress can promote recovery. Such programmatic approaches likely accelerate improvements by normalizing participation in meaningful life roles throughout treatment.

Evaluating the effects of interventions on perceived autonomy support and psychological growth can enable services to adapt treatment approaches to improve outcomes. Using brief, validated tools such as the HCCQ and MHRM alongside traditional symptom and functioning metrics can guide quality-improvement initiatives, prioritize autonomy support and psychological growth as key treatment targets, and help communicate their importance to both providers and patients. Embedding these evaluative practices within early psychosis programs ensures that autonomy support becomes a targeted and actionable dimension of care, integrated into pharmacological, psychosocial, and vocational interventions. This approach offers a framework for strengthening motivation, engagement, collaboration, and long-term recovery outcomes.

### Study Strengths

A major strength of this study is the use of a large, diverse, longitudinal dataset that enables robust analysis of treatment processes and outcomes. The RAISE-ETP design, with its clear contrast between the NAVIGATE intervention and standard care, offers a unique opportunity to evaluate autonomy-supportive interventions in real-world settings. Our integration of self-report measures of autonomy support and recovery-oriented psychological growth with clinician-rated assessments of functioning provides a rigorous framework for linking subjective experiences to objective outcomes. Relatedly, use of the MHRM further strengthens the study by capturing recovery processes grounded in service-user perspectives, consistent with PROMs approaches^54^^,^^55^ that ensure measures administered resonate with the actual challenges and recovery trajectories of the population studied.

### Study Limitations and Future Directions

This study is not without limitations. A primary constraint is the inherent nature of mediation models, which, despite robust analytical strategies, can only imply rather than confirm causality. Our attempt to employ cross-lagged panel analysis to bolster causal inference yielded poor fit, reflecting broader challenges in modeling measurement timing. Measurement timing is often more influenced by logistical feasibility than by theoretical guidance.^56–58^ The temporal lag between autonomy support and recovery-oriented psychological growth, and between recovery-oriented psychological growth and functional improvements is not known. More frequent assessment would allow growth curve analyses to be developed that would better inform the timing of measurement for mediational studies. Repeated measurements would also enable use of models that explicitly capture within-person change, such as latent growth curve mediation and multilevel (time-varying) mediation models. By providing granular data on the effects of autonomy support over time, researchers could gain clearer insights into optimal intervention timings to refine therapeutic strategies and maximize treatment outcomes. Engagement across NAVIGATE components (eg, supported employment, family psychoeducation) likely influenced autonomy support and psychological growth. Although engagement was not directly analyzed, variation may explain individual differences; future research integrating treatment dose and qualitative data could clarify these effects. Finally, although we estimated mediation models separately by condition due to prior evidence of divergent trajectories in autonomy support, this approach precluded a formal test of whether mediation paths differed significantly across groups. Future research with adequate power could evaluate moderated mediation models to directly assess group-level differences in structural pathways.

## Conclusion

In summary, this study delineates the psychological mechanisms mediating the association between perceived autonomy support and functional outcomes in FEP and SSDs, highlighting the central role of recovery-oriented psychological growth in this process. Leveraging the rigor and scope of the RAISE-ETP dataset, the findings demonstrate that the NAVIGATE program—by embedding SDT principles within coordinated specialty care—facilitates not only improvements in functioning but also the internal growth processes identified by patients as central to recovery, as captured by the MHRM. Collectively, these results extend current evidence on autonomy-supportive care by indicating that comprehensive, system-level implementation of autonomy-supportive practices fosters enduring psychological growth, agency, and quality of life.

## Data Availability

This study involved secondary analysis of data from the Recovery After an Initial Schizophrenia Episode–Early Treatment Program (RAISE-ETP). Access to the dataset was granted following approval of a data use request submitted to the National Institute of Mental Health (NIMH) Data Archive. The data are publicly available to qualified researchers through the NIMH Data Archive (https://nda.nih.gov/) upon submission and approval of a data use application.
